# Évaluation du psycho-traumatisme lié aux agressions commises par les patients psychotiques sur leurs aidants familiaux: enquête transversale dans la région de Sfax, Tunisie

**DOI:** 10.11604/pamj.2021.39.266.25413

**Published:** 2021-08-24

**Authors:** Nada Charfi, Maroua Daoud, Sana Omri, Najeh Smaoui, Rim Feki, Jihène Ben Thabet, Lobna Zouari, Manel Maâlej Bouali, Mohamed Maâlej

**Affiliations:** 1 Service de Psychiatrie C, Centre Hospitalier Universitaire de Hédi Chaker, Sfax, Tunisie,; 2 Faculté de Médecine de Sfax, université de Sfax, Sfax, Tunisie

**Keywords:** Agression, aidant, état de stress, post-traumatique, prévention, psychose, Aggression, caregiver, post-traumatic stress disorder, prevention, psychosis

## Abstract

**Introduction:**

prendre en charge un patient psychotique est associé à un risque d´exposition à la violence qui peut toucher tous les intervenants, en particulier l´entourage familial. La violence que la fonction d´aide engendre peut avoir un impact psychologique sur l´aidant. Nos objectifs étaient de relever la prévalence des agressions perpétrées par les patients psychotiques envers leurs aidants familiaux, évaluer l´impact psycho-traumatique chez eux et identifier les facteurs qui lui sont associés.

**Méthodes:**

des aidants familiaux de patients psychotiques ont été interviewés. Nous avons utilisé deux échelles psychométriques: l´échelle de perception de la prévalence de l´agression et l´échelle révisée d´impact de l´événement.

**Résultats:**

le nombre total des participants était de 95. Trois aidants sur quatre (75,8%) ont rapporté avoir été victimes d´agression modérée à sévère de la part de leurs proches malades durant l´année écoulée. Une agression modérée à sévère était significativement plus fréquente chez les aidants de sexe masculin, d´un âge plus avancé, parents de patient psychotique et vivant dans un autre foyer que lui. Un taux de 54,7% des aidants présentait un état de stress post-traumatique (ESPT) probable et le risque d´apparition de ce trouble augmentait significativement avec l´intensité perçue des agressions. Le même profil sociodémographique de l´aidant lié à la gravité des agressions était à risque de développement d´un ESPT chez l´aidant.

**Conclusion:**

les agressions commises par les patients psychotiques sur leurs aidants familiaux paraissent avoir un impact psycho-traumatique important. Aussi, des interventions ciblant la violence des malades psychotiques dans leur milieu familial devraient être intégrées dans la prise en charge.

## Abstract (en)

**Introduction:**

the management of patients with psychosis is associated with a risk of exposure to violence which can affect all the stakeholders, in particular the family entourage. Caregiving role generates violence and this can have a psychological impact on the caregivers. The purpose of this study is to evaluate the prevalence of aggressions perpetrated by patients with psychosis on their family caregivers as well as to assess the psychological and traumatic impact on family caregivers and to identify factors associated with it.

**Methods:**

family caregivers of patient with psychosis were interviewed. We used two psychometric scales: the perception of prevalence of aggression scale (POPAS) and the impact of event scale-revised.

**Results:**

the whole number of participants was 95. Three out of four caregivers (75.8%) reported having been subjected to moderate to severe aggressions by their sick relatives during the past year. Moderate to severe aggressions were significantly more common among older male caregivers, parents of patients with psychosis living in a different home. Fifty-four point seven percent of caregivers had possible posttraumatic stress disorder (PTSD) and the risk of developing this disorder increased significantly as the perceived severity of aggressions increased. The same sociodemographic profile of the caregivers, related to the severity of the aggressions, was associated with a risk of developing PTSD in the caregivers.

**Conclusion:**

the aggressions committed by patients with psychosis against their family caregivers appear to have a significant psychological and traumatic impact. Interventions targeting violence committed by patients with psychosis in their family environment should also be integrated into patient management.

## Introduction

La prise en charge des patients présentant des troubles psychotiques est complexe. L´entourage familial y joue un rôle fondamental; il constitue un élément clé dans l´aide apportée à ces patients. Cette relation d´aide n´est pas dénuée de risque pour les aidants. En effet, au cours de la fonction d´aide, plusieurs tensions peuvent être mises en jeu entre aidés et aidants familiaux, et peuvent générer de l´agressivité. Un tel trouble du comportement est fréquemment observé au cours de l´évolution de la psychose et les aidants en constituent des victimes potentielles.

La violence subie inhérente au rôle d´aidant d´un patient psychotique, peut entraîner une altération de la santé psychologique de l´aidant. En effet, la violence interpersonnelle est considérée par l´Organisation Mondiale de la Santé comme un problème de santé publique pouvant avoir un impact significatif sur le bien-être de la personne [1]. Sur le plan individuel, les troubles psychiatriques en particulier la dépression et l´anxiété sont significativement plus fréquents chez les aidants comparativement à la population générale [2]. En outre, les aidants peuvent développer des symptômes de stress post-traumatique liés à leur exposition aux comportements violents que la prestation de soins engendre.

Les stratégies de prévention de la violence au sein du milieu familial des patients psychotiques devraient en premier lieu assurer une analyse et une compréhension de l´expérience subjective des aidants victimes d´agression et son impact psychologique sur eux. Aussi, nous avons mené cette étude dans le but d´évaluer la perception, par les aidants des patients psychotiques, de la prévalence des agressions commises par ceux-ci contre eux, et d´évaluer l´impact psycho-traumatique chez eux et d´identifier les facteurs qui lui sont associés.

## Méthodes

**Type et lieu de l´étude:** il s'agissait d'une étude transversale, descriptive et analytique. Elle s´est déroulée au service de psychiatrie au Centre Hospitalo-universitaire Hédi Chaker de Sfax (Tunisie).

**Population d´étude:** nous avons recruté les aidants familiaux de patients présentant un trouble psychotique chronique à type de schizophrénie, de trouble schizo-affectif ou de trouble bipolaire. Ce diagnostic a été établi par un psychiatre en référence aux critères diagnostiques du manuel diagnostique et statistique des troubles mentaux dans sa 5^e^ édition (DSM-5). Les aidants se sont présentés avec le patient soit à l´unité des consultations externes dans le cadre du suivi en ambulatoire, soit à l´unité d´hospitalisation lors d´un séjour hospitalier du malade. Nous avons expliqué aux aidants le cadre et les objectifs de l´étude et insisté sur l´anonymat et la confidentialité des données recueillies. Ils ont été inclus dans l´étude après un consentement oral éclairé.

**Les critères d'inclusion** de l´aidant étaient les suivants: a) l´âge supérieur ou égal à 18 ans; b) ayant un lien de parenté avec le patient de type conjoint, ascendant, descendant ou collatéral (frère/sœur); c) être fortement impliqué dans la gestion des troubles liés à la maladie du proche et un contact régulier avec le patient d´au moins dix heures par semaine ; ayant donné un consentement libre et éclairé pour participer à l´étude.

**Les critères de non-inclusion** étaient: a) l´âge inférieur à 18 ans; b) le contact rare avec le patient estimé à moins de dix heures par semaine; c) l´exposition antérieure à des facteurs stressants majeurs ou traumatisants; d) la présence d´une histoire personnelle de trouble psychiatrique; e) l´absence de consentement.

**Recueil des données:** la fiche utilisée pour le recueil des données comportait deux parties: l´une pour recueillir, suite à un entretien, les données relatives au profil de l´aidant (lien de parenté avec le patient, sexe, âge, statut marital, statut professionnel, lieu de vie et durée de la fonction d´aide), l´autre réservée aux données relatives au profil du patient (âge, sexe, statut marital, activité professionnelle, diagnostic clinique) recueillies à l´aide d´un entretien avec le patient et à partir de son dossier médical.

Nous avons assuré une hétéro-passation de deux échelles psychométriques pour chacun des aidants.

**L´échelle de perception de la prévalence de l´agression (PAOPS):** ce questionnaire est conçu initialement pour mesurer la fréquence d´exposition à des comportements agressifs de patients, perçue par le travailleur du milieu de la santé et des services sociaux au cours des 12 derniers mois [3]. Par la suite, l´étude de Loughland *et al*. [4] en Australie a appliqué le POPAS aux proches aidants. Loughland *et al*. ont proposé quatre facteurs: le facteur « violence verbale » composé de la violence verbale indirecte, la menace verbale, l´humiliation, la provocation et les comportements passifs-agressifs, le facteur « violence physique » regroupait l´intimidation physique, la violence sur le matériel, la violence physique sans blessure ou causant des blessures mineures et la violence physique causant des blessures graves, le facteur « violence envers soi-même» comprenait l´automutilation causant des blessures mineures, l´automutilation causant des blessures graves, la tentative de suicide et le suicide et le facteur « violence sexuelle » comprenait le harcèlement sexuel et l´agression sexuelle. Pour chacun de ces types de violence, les répondants doivent inscrire sur une échelle de type Likert à cinq points (« jamais », « occasionnellement », « parfois », « souvent », « fréquemment ») la fréquence perçue de leur propre exposition dans les 12 derniers mois. Le score correspondant à chaque facteur est obtenu par la division du total des scores de chaque item par le nombre d´items. Le profil de la POPAS totale comprend deux sous-groupes: agression absente/ légère et agression modérée/ sévère. Le dernier sous-groupe correspond à ceux ayant une agression modérée/ sévère à l´un des quatre facteurs au moins. La POPAS est reconnu comme un instrument fidèle. Les études qui l´ont utilisé ont obtenu des alphas de Cronbach allant de 0,70 à 0,90 [5-7]. Dans la présente étude, nous avons utilisé la version canadienne en langue française validée [8].

**L´échelle révisée d´impact de l´événement (IES-R):** créée par Weiss et Marmar en 1996, elle a été construite à partir de l´échelle de l´impact de l´événement d´Horowitz *et al*. (1979) [9], et traduite et validée en français par Brunet et al. en 2003 [10]. L´IES-R mesure la présence de stress traumatique lié à des événements violents. L´échelle est composée de 22 items qui évaluent l´intensité de chaque symptôme, sur une échelle de Likert allant d´«extrêmement: 4» à «pas du tout: 0». Elle comprend huit items pour les symptômes de reviviscence, huit items pour les symptômes d´évitement et six items pour les symptômes d´hyperactivité neuro-végétative. Un score total est obtenu en additionnant les différents scores pour chaque item. D´après les auteurs, un score de 36 suggérerait la présence d´un état de stress post-traumatique (ESPT), sans toutefois poser un diagnostic.

**Analyse des données:** la saisie et l´analyse des données ont été réalisées en utilisant le logiciel informatique *Statistical Package for Social Sciences (SPSS)* dans sa 20^e^ version. Nous avons calculé des fréquences absolues et des fréquences relatives (pourcentages) pour les variables qualitatives. Nous avons calculé des moyennes et des écarts-types pour les variables quantitatives. Les comparaisons de pourcentages sur séries indépendantes ont été effectuées par le test de chi-deux de Pearson, et en cas de significativité au test de chi-deux et de non-validité de ce test (si les effectifs théoriques du tableau de contingence sont < 5), la comparaison a été faite par le test exact bilatéral de Fisher. Pour la comparaison des moyennes des variables quantitatives, nous avons utilisé le test t de *Student*. Le seuil de significativité a été fixé à une valeur p inférieure ou égale à 0,05.

## Résultats

Le nombre total de participants inclus dans notre population d’étude était ainsi de 95.

**Description de la population d´étude:** l´âge moyen des aidants était de 47,71 ans (ET=12,96 ans) avec 51,6% d´hommes et 48,4% de femmes. Le lien de parenté avec l´aidé était un conjoint (42,1%), un parent (26,3%), un enfant (5,3%) ou un frère ou sœur (26,3%). Ils vivaient dans le même foyer que le patient dans 71,6% des cas. Leur niveau socioéconomique était bas dans 47,4% et moyen dans 52,6% des cas. Les aidants ont estimé que le nombre de jours par mois en contact avec le patient était de 27,7 jours en moyenne (ET= 12,96 jours). Les aidés avaient un âge moyen de 38,81 ans (ET=5,65 ans). Ils étaient de sexe masculin dans 67,4% des cas, non mariés dans 53,7% des cas et inactifs sur le plan professionnel dans 55,8% des cas. Ils étaient atteints d´un trouble bipolaire dans 43,2% des cas et d´une schizophrénie dans 56,8% des cas.

**Evaluation de l´exposition des aidants aux actes d´agression par le PAOPS:** la violence verbale était le type d´agression le plus rapporté par les aidants (score moyen=2,66) suivie par la violence physique (score moyen=2,15), puis la violence envers soi-même (score moyen=1,21) et la violence sexuelle (score moyen=1,12) (Tableau 1). Selon le PAOPS total, l´intensité des actes d´agression était modérée à sévère dans 75,8% des cas (n=72).

**Tableau 1:** scores moyens des différents items de la PAOPS

Variables	Moyenne	Ecart-type
**Facteur «violence verbale»**	2,66	0,78
**violence verbale indirecte**	2,95	1,34
**menace verbale**	3,38	1,07
**humiliation**	2,35	1,17
**provocation**	2,43	0,88
**comportements passifs-agressifs**	2,20	1,08
**Facteur «violence physique»**	2,15	0,66
**intimidation physique**	2,02	1,18
**violence sur le matériel**	2,57	1,36
**violence physique mineure**	2,80	0,92
**violence physique grave**	1,24	0,56
**Facteur «violence envers soi-même»**	1,21	0,30
**auto-agressivité causant des blessures mineures**	1,27	0,59
**auto-agressivité causant des blessures graves**	1,18	0,43
**tentative de suicide**	1,40	0,70
**suicide**	1,00	0,00
**Facteur «violence sexuelle»**	1,12	0,33
**harcèlement sexuel**	1,25	0,66
**agression sexuelle**	1,00	0,00

**PAPOS:** perceptions of prevalence of aggression scale

**Evaluation de l´impact psycho-traumatique des actes d´agression chez les aidants:** le score total moyen des aidants à l´échelle IES-R était de 57,35 ±32,93. Les scores moyens des trois dimensions étaient comme suit: symptômes de réviviscence: 21,04±12,22 symptômes d´évitement: 19,13±12,41, symptômes d´hyperactivité neurovégétative: 17,16± 8,60. Un ESPT était probable chez 52 aidants (54,7%). Les corrélations entre les facteurs sociodémographiques des aidants et la perception de la prévalence de l´agression sont présentées dans le Tableau 2. Chez les aidants qui ont été exposés à une agression modérée à sévère (n=72/95), un ESPT probable était plus fréquent chez ceux de sexe masculin, parents (père ou mère), ne vivant pas dans le même foyer que le patient et de niveau socioéconomique moyen. L´âge de l´aidant et la durée de la fonction d´aide étaient significativement plus élevés en cas d´ESPT (Tableau 3). L´ESPT était significativement plus fréquent en cas d´exposition de l´aidant à une agression modérée à sévère (70,8% versus 4,3%; p=0,00). Chez les aidants exposés à une agression modérée à sévère (n=72/95), les scores moyens des facteurs de violences verbale, physique, envers soi-même et sexuelle étaient significativement plus élevés en cas d´ESPT (Tableau 4).

**Tableau 2:** corrélations entre les caractéristiques des aidants et leurs perceptions de l’intensité de l’agression (n=95)

	Intensité de l´agression		p
	Absente/légère	Modérée/sévère	
**Moyenne d´âge en an (ET)**	41,30(3,86)	49,75(14,15)	<0,001
**Sexe**			<0,001
Masculin	2%	98%	
Féminin	47,8%	52,2%	
**Lien de parenté**			0,001
Parents	0%	100%	
Autre	32,9%	67,1%	
**Lieu de vie**			0,001
Dans le même foyer que le patient	33,8%	66,2%	
Dans un autre foyer	0%	100%	
**Niveau socioéconomique**			
Bas	28,9%	71,1%	0,310
Moyen	20%	80%	
**Durée de la fonction d´aide/ mois en jours (ET)**	30(0,00)	27,13(6,95)	0,001

**ET**: écart-type

**Tableau 3:** corrélations entre les caractéristiques des aidants et la présence ou non d´un état de stress post-traumatique (n=72)

	Groupes selon IES-R		
Variables	absence d´ESPT	ESPT probable	p
**Moyenne d´âge (ET)**	38,67 ans(4,53)	54,31 ans(14,26)	<0,001
**Sexe**			<0,001
Masculin	6,2%	93,8%	
Féminin	75%	25%	
**Lien de parenté**			<0,001
Parents	0%	100%	
Autre	44,7%	55,3%	
**Lieu de vie**			<0,001
Dans le même foyer que le patient	44,4%	55,6%	
Dans un autre foyer	3,7%	96,3%	
**Niveau socioéconomique**			0,003
Bas	46,9%	53,1%	
moyen	15%	85%	
**Durée moyenne de la fonction d’aide/ mois (ET)**	23,00 jours (10,24)	28,82 jours(4,07)	0,010

**ESPT**: état de stress post-traumatique**; ET:** écart-type; **IES-R**: échelle révisée d´impact de l´événement

**Tableau 4:** scores moyens des facteurs d’agression chez les aidants exposés à des agressions modérées à sévères selon la présence ou non d’un ESPT (n=72)

	Groupes selon IES-R		
Scores moyens POPAS	ESPT absent	ESPT probable	p
facteur «violence verbale»	2,63 (ET=0,28)	3,18(ET=0,49)	0,00
facteur «violence physique»	1,55 (ET=0,37)	2,65(ET=0,38)	0,00
facteur «violence envers soi-même»	1,01(ET=0,05)	1,24(ET=0,29)	0,00
facteur «violence sexuelle»	1,00 (ET=0,00)	1,23 (ET=0,42)	0,00

**ESPT:** état de stress post-traumatique; **ET:**écart-type; **IES-R**: échelle révisée d´impact de l´événement; **PAPOS:** perceptions of prevalence of aggression scale

## Discussion

Dans notre étude, approximativement trois aidants sur quatre (75,8%) ont rapporté avoir été victimes d´actes d´agression d´intensité modérée à sévère de la part de leurs proches malades durant l´année écoulée. Cette estimation dépasse celles rapportées dans la littérature. Selon Hayes *et al*. [2], les taux d´exposition à la violence des patients psychotiques variaient d´une étude à une autre mais ils oscillaient globalement entre 50 et 60% et dans l´étude de Kageyama *et al*. [11] environ un tiers des aidants ont signalé des incidents d´agression durant l´année précédant l´évaluation. Cependant, la comparaison avec les autres études doit considérer l´absence d´homogénéité des définitions, des outils de mesure des agressions et des périodes d´évaluation sur lesquelles elles se sont basées. En ce qui concerne la mesure des actes d´agression, dans certaines études, le recueil des données s´est fait à partir des dossiers médicaux des patients et des échelles d´évaluation des symptômes cliniques, alors que dans d´autres, il s´est basé sur les auto-questionnaires auto-rapportés ou les interviews directes avec les aidants [12]. Dans notre étude, la méthode utilisée pour la détermination de la prévalence des incidents d´agression rapportés par les aidants aurait pu induire une surestimation, pour mettre en exergue leur niveau de fardeau élevé. Les aidants auraient pu aussi sous-estimer la prévalence des actes d´agression, pouvant être plus ou moins attribués à leur maladresse dans l´approche du malade et donc à leur incompétence dans la fonction d´aide.

La violence à l´encontre de l´aidant peut revêtir diverses expressions. Chez les aidants interviewés, les violences verbale et physique étaient les plus fréquentes avec des scores moyens au niveau du PAPOS de 2,66 et 2,15 respectivement. De façon concordante avec les données de la littérature, la violence verbale était la plus fréquemment rapportée par les aidants (score moyen au POPAS le plus élevé). Ces actes d´agression verbale étaient le plus souvent perçus par les aidants comme étant effrayants et menaçants, du fait qu´ils constituaient une menace pour leur intégrité physique ou une menace verbale claire de représailles envers-eux ou les autres proches (score moyen PAPOS le plus élevé: 3,38). Nijman *et al*. ont également trouvé dans leur étude multicentrique menée dans quatre hôpitaux psychiatriques à Londres que 80 à 90% des soignants ont subi des violences verbales menaçantes ou non dans l´année précédant l´étude [3]. Dans la même étude, la violence physique vécue par les aidants était d´intensité modérée à sévère dans 63,2% des cas; il s´agissait de coups de pieds, de frapper, de pousser, ou de comportements agressifs destructeurs envers le matériel comme la dégradation des biens d´autrui, des biens personnels ou bris d´objets. Les aidants ont été aussi confrontés, mais avec une fréquence moindre, à l´auto-agressivité des malades psychotiques ayant causé des blessures, et à des tentatives de suicide. De tels actes sont fréquemment observés chez les malades psychotiques et constituent souvent un motif d´hospitalisation en milieu psychiatrique. L´exposition à la violence sexuelle était rarement rapportée par les aidants et quand elle était présente, elle était d´intensité légère. Cette violence était à type de tentative d´attentat à la pudeur sans jamais atteindre le degré d´agression sexuelle. Toutefois, il est à noter que la violence sexuelle demeure un sujet tabou et donc le plus souvent dissimulée dans notre société, a fortiori quand l´agresseur appartient à la même famille.

Les aidants d´un âge plus avancé étaient significativement plus fréquemment agressés par les patients psychotiques (p=0,00). Ce résultat pourrait être expliqué par le fait que les aidants jeunes sont capables de mieux se défendre et se protéger, contrairement aux aidants plus âgés. Par ailleurs, les aidants de sexe féminin étaient moins fréquemment exposés aux actes d´agression que ceux de sexe masculin; ce qui donne à penser que les femmes ont une meilleure gestion de la relation avec de tels malades ou bien qu´elles sont moins sollicitées que les hommes dans les situations susceptibles de générer de la violence. Dans notre étude, le risque d´actes d´agression était aussi plus élevé chez les aidants vivant dans un foyer autre que celui du patient (p=0,001). Alors que plusieurs études antérieures ont trouvé que la cohabitation était un facteur de risque d´exposition de l´aidant à la violence du patient [13,14]. La répartition disproportionnée des aidants selon leur lieu de vie et la prédominance de ceux vivant dans le même foyer que le patient (71,6%) pourrait expliquer en partie la divergence entre ce résultat et les données de la littérature. De plus, le fait de vivre loin du patient et de ne pas être disponible tout le temps pour lui, pourrait engendrer des sentiments d´hostilité, contexte qui pourrait faire le lit à des réactions violentes.

Les parents étaient significativement plus fréquemment exposés aux actes d´agression de la part des patients que les conjoints, les enfants ou la fratrie (p=0,001). Dans ce sens, Fawzi *et al*. ont noté dans une étude menée en Egypte auprès des patients présentant un premier épisode psychotique, que 40% ont commis des abus physiques à l´encontre de leurs parents durant les deux mois précédant la consultation [15]. Ce résultat est a priori surprenant eu égard aux valeurs de la société arabo-musulmane qui accordent une place privilégiée aux ainés et, a fortiori, aux parents; ceux-ci devraient être écoutés, chouchoutés voire vénérés. Aussi, la maltraitance des parents et surtout la violence à leur encontre constituent un péché majeur, impardonnable. Cependant, ici les agresseurs sont des malades psychotiques, dont les capacités de jugement et de discernement sont abolies. De plus, la proximité avec les parents et la fréquence des délires à thème de persécution impliquant très souvent les proches et, en particulier, les parents accroissent le risque d´apparition des comportements agressifs.

Les aidants qui assuraient la fonction d´aide de façon quotidienne étaient significativement moins exposés à l´agression des patients (p=0,001). A ce propos, il convient de noter que les membres de la famille qui sont présents tout le temps auprès du malade et s´impliquent dans le processus de soins en lui apportant un soutien lors des crises, parviennent à mieux gérer les changements de son comportement. Cet investissement de la famille dans les soins des personnes affectées permettrait de prévenir ou, du moins, de réduire le risque d´actes agressifs. Dans notre étude, l´évaluation de l´ESPT par l´IES-R a montré que 54,7% des aidants présentaient un ESPT probable et que le risque d´apparition de ce trouble augmentait significativement avec l´intensité perçue des agressions (p<0,001). Cette prévalence de l´ESPT trouvée dans la population des aidants est nettement supérieure à celle trouvée dans la population générale aux Etats-Unis, qui est de 3,5% [16]. En fait, les données de la littérature mettent en exergue une association entre la violence des patients psychotiques et les symptômes post-traumatiques chez les aidants. Une étude australienne menée par Loughland *et al*. [4] auprès de 155 proches de patients atteints de schizophrénie, a trouvé que 77% des aidants rapportaient des expériences d´agressions modérées à sévères de la part des patients et que 51,5% d´entre eux présentaient un ESPT caractérisé. Dans le même sens, Hanzawa *et al*. [17] ont trouvé que les aidants des patients atteints de schizophrénie agressifs au quotidien, présentaient des niveaux plus élevés de reviviscence (9,46±6,78), d´évitement (10,32±6,83) et d´hyperréactivité (6,91±5,59) à l´IES-R, en rapport avec un ESPT. Dans notre étude, les scores moyens des trois dimensions de l´ESPT moyennant la même échelle (21,04±12,22; 19,13±12,41; 17,16±8,6 respectivement) étaient plus élevés que ceux rapportés par Hanzawa *et al*. témoignant de l´intensité de la symptomatologie chez les aidants de notre étude victimes de violence.

La prévalence relativement élevée de l´ESPT dans notre population d´étude aurait un certain rapport avec le moment de l´évaluation qui coïncidait, pour certains aidants, avec l´hospitalisation de leur proche malade. Ceux-là auraient vécu des moments de crise avant l´hospitalisation du patient, suite à la décompensation de sa psychose, avec souvent des troubles graves du comportement engendrant un risque d´exposition important à des évènements traumatiques. Un tel contexte constitue un facteur de risque d´apparition d´un ESPT. Dans notre étude, les scores PAPOS des quatre facteurs de violence (verbale, physique, envers soi-même ou sexuelle) étaient significativement plus élevés dans le groupe des aidants avec un ESPT. Ainsi, l´association entre l´ESPT et l´exposition aux agressions était indépendante du type d´agression. Alors que dans l´étude menée au Japan par Kageyama M *et al*. [18] auprès de 353 parents de patients atteints de schizophrénie, seule l´exposition à la violence physique engendrant des blessures graves de la part de leurs enfants malades était susceptible de se compliquer d´un ESPT (OR= 2,03). A ce propos, rappelons que dans le DSM-5, le diagnostic de trouble post-traumatique suppose une confrontation brutale et directe à la mort ou à une menace de mort ou à une scène horrifiante comme les violences sexuelles ou les blessures graves pour soi ou pour autrui [19]. Ceci pourrait expliquer le risque d´apparition d´un ESPT chez les aidants suite à leur confrontation directe à une violence physique ou sexuelle ou à une menace de mort ou d´agression proférée verbalement par le patient ou en étant témoins directs d´une agression perpétrée par le patient contre soi-même comme les tentatives de suicide ou les automutilations avec des lésions graves.

Chez les aidants de notre étude qui ont été exposés à des agressions modérées à sévères (n=72/95), l´âge avancé (p<0,001), le genre masculin (p<0,001), le lien de parenté de type parent-enfant (p=0,00) et le fait de vivre dans un foyer différent de celui du patient (p<0,001) ressortaient comme des facteurs associés à l´apparition d´un ESPT. Dans la population générale, c´est plutôt le genre féminin qui serait associé à une plus grande vulnérabilité de développer un ESPT du fait d´une différence au niveau des évènements traumatiques vécus; les femmes sont plus susceptibles de subir des agressions sexuelles alors que les hommes subissent davantage d´agressions physiques [20]. Ces résultats donnent à penser que c´est l´évènement traumatique en soi, plutôt que les caractéristiques sociodémographiques des aidants qui pourraient induire une plus grande vulnérabilité à l´ESPT. Cela s´explique entre autres par le fait que les données sociodémographiques impliquent souvent la présence de variables concomitantes qui peuvent par elles-mêmes influencer ou même expliquer la vulnérabilité ou la résilience des aidants. En effet, l´épuisement et la détresse psychologique des aidants familiaux en rapport avec l´ESPT entraînent une atteinte de l´intégrité du système familial du fait de la présence de troubles qui menacent son équilibre. Ceci pourrait les affecter et les empêcher de s´acquitter de leurs fonctions essentielles vis-à-vis de leurs proches malades, en particulier le soutien à l´observance du traitement et l´adhésion au processus de soins.

## Conclusion

Dans la présente étude, les actes d´agression modérés à sévères ressortaient comme un facteur lié à l´augmentation du risque d´apparition d´un ESPT chez les aidants familiaux des patients psychotiques. Il serait alors pertinent de repérer les familles confrontées à de tels phénomènes, afin de mettre en œuvre un suivi familial et répondre aux besoins spécifiques des aidants en tenant compte des facteurs de risque identifiés. Les professionnels de la santé mentale devraient être sensibilisés au problème d´exposition à la violence des aidants familiaux de patients psychotiques et à la nécessité de l´intégrer dans les schémas thérapeutiques. Ainsi, il serait pertinent d´évaluer systématiquement le risque d´agression en intra-familial, lors des hospitalisations ou du suivi en ambulatoire. Des groupes d´éducation et de soins familiaux peuvent renforcer les compétences des aidants pour repérer et désamorcer les situations de violence et agir sur les éventuels facteurs facilitants. Pour cela, des interventions ciblant la gestion de la violence et la résolution de problèmes sont utiles pour prévenir l´apparition d´une pathologie post-traumatique.

### Etat des connaissances sur le sujet

- Prendre en charge un patient psychotique est associé à un risque d´exposition à la violence qui peut toucher tous les intervenants, en particulier l´entourage familial;
- La violence subie inhérente au rôle d´aidant d´un patient psychotique, peut entraîner une altération de la santé psychologique de l´aidant.

### Contribution de notre étude à la connaissance

- Trois aidants sur quatre étaient victimes d´agressions modérées à sévères de la part de leurs proches psychotiques durant l´année écoulée;
- La violence que la fonction d´aide engendre était associée à un état de stress post-traumatique probable dans plus de la moitié des cas;
- La vulnérabilité à l´état de stress post-traumatique chez les aidants était plutôt liée à l´évènement traumatique en soi qu´à leurs profils sociodémographiques.
